# Impediments and catalysts to task-shifting psychotherapeutic interventions for adolescents with PTSD: perspectives of multi-stakeholders

**DOI:** 10.1186/s13034-017-0187-y

**Published:** 2017-09-22

**Authors:** Tanya van de Water, Jaco Rossouw, Elna Yadin, Soraya Seedat

**Affiliations:** 1 Department of Psychiatry, Stellenbosch University, Cape Town, South Africa; 2 Department of Psychiatry, University of Pennsylvania, Philadelphia, PA USA; 3 Faculty of Medicine and Health Sciences, Stellenbosch University, PO Box 241, Cape Town, 8000 South Africa

**Keywords:** Task-shifting, South Africa, Adolescents, Nurses, School, PTSD, Barriers, Facilitators

## Abstract

**Background:**

This qualitative study was nested within a randomized controlled trial (RCT) where two psychotherapeutic interventions (supportive counselling and prolonged exposure for adolescents) were provided by supervised nurses (who served as ‘nurse counsellors’) to adolescents with PTSD in school settings. This paper describes the perspectives of nurse counsellors (NCs) and school liaisons (SLs). SLs were teachers or administrative personnel at the schools who coordinated the study visits of participants with the NCs. We focus on the impediments and catalysts to and recommendations for treatment implementation.

**Methods:**

NCs (n = 3) and SLs (n = 3) who participated in the RCT during 2014 were purposively recruited by telephone and participated in face-to-face semi-structured in-depth interviews that were recorded and doubly transcribed. Thematic content analysis was applied using Atlas.ti software to identify emerging themes. This paper describes the impediments and catalysts to provide psychotherapy by task-shifting in a community setting across three sub-themes: personal, community, and collaborative care.

**Results:**

Although nurses were initially resistant to supervision it was central to personally coping with complex interventions, managing traumatic content, and working apart from a multi-disciplinary team. Delivering the interventions in the community presented multiple logistical impediments (e.g. transport, communication, venue suitability) which required creative solutions. In light of resource shortages, networking is central to effective delivery and uptake of the interventions. Collaboration between government departments of health and education may have a major impact on providing school-based psychotherapy through task-shifting.

**Conclusions:**

Impediments to implementation are not insurmountable. This article provides recommendations to maximize the success of task-shifting interventions should they be rolled out.

## Background

Task-shifting is the rational redistribution of tasks among health teams and involves the appropriate transfer of specific tasks from specialists to those with abbreviated training [1]. Because of resource shortages, this approach has risen in popularity in the treatment of various health problems including tuberculosis [2–4], HIV [5–7], midwifery [8, 9], and mental health [10–14]. Task-shifting has been used to treat PTSD: (i) in adult refugees placed in Uganda by applying narrative exposure therapy and trauma counselling [15], (ii) adult survivors of systematic violence in Thailand and Iraq using Common Elements Treatment Approach (CETA) [16], (iii) adult survivors of torture in Iraq using Cognitive Processing Therapy and CETA in community settings [17], and (iv) in orphaned and vulnerable children in Zambian community settings using trauma focused cognitive behaviour therapy (TF-CBT) [18]. These studies did not report on the in depth experiences of the non-specialist health workers who provided the task-shifted interventions.

Other studies report that task-shifting implementation can be hindered by professional and institutional resistance (e.g. seeing task-shifting as competition), lack of regulatory frameworks, limited funding, concerns about the quality of care (e.g. not acting in close consultation with specialist) [15], interruptions, lack of privacy, and costs of ongoing expert training and supervision [12]. The efficacy of task-shifting is dependent on training, supervision, support and teamwork, regardless of field [8, 14, 16]. Practical training is the most effective way to train new skills and to reduce the anxiety of undertaking complex new responsibilities [8, 12]. For example, community mental health workers and stakeholders in Ghana highlighted the need to review task-shifting roles, training, and supervision arrangements to ensure quality of care [19].

## Objectives

No existing studies exclusively provide qualitative descriptions of the barriers, facilitators, and recommendations for implementation of task-shifting in a community based setting from the perspectives of non-specialist health workers. We sought to explore the impediments and catalysts to and recommendations for the implementation of psychotherapeutically informed task-shifting interventions, through the lenses of NCs and SLs. Their perspectives are important when considering rapid scale-up of these evidence-based interventions in community settings.

## Methods

### Framework

We undertook a nested, qualitative study evaluating the experiences of stakeholders who participated in a randomized controlled trial (RCT) [20]. We used a biomedically influenced empiricist framework, which places emphasis on biomedical causal mechanisms, evidence-based practices and measurable outcomes. Both first and second authors are practicing clinical psychologists from an upper-middle class background and, as such, the data was not approached in a value-free manner. Instead, the authors utilized their knowledge obtained through clinical practice. In order to neutralize the researchers’ inherent investment in the success of the task-shifting paradigm, an empiricist framework was adopted. The authors were concerned with uncovering the truth and presenting it through empirical means [21], believing knowledge to be hard, real, and acquirable. Thus a systematic methodology that relied on control was employed in data analysis.

### RCT sampling

For the above mentioned RCT [22], registered nurses studying towards a diploma in advanced psychiatry were trained (5 days of theoretical and practical application) in prolonged exposure therapy for adolescents (PE-A) [25–25] and supportive counselling (SC) [26].

In 2014, six nurses volunteered to participate in the study as an opportunity to add variety to their course prescribed practical hours. All six met the inclusion criteria (doing an advanced nursing certificate in psychiatry and having their own transport) and received the training. NCs were allocated to treatment and participant through block randomization. As such, NCs were randomly assigned to PE-A and SC cases. The nurses received weekly group supervision with the trainer (second author is a male psychologist, experienced PE-A and SC therapist and supervisor, study PI) where they discussed video recordings of both PE-A and SC cases.

The interventions were provided at participating schools, except during school holidays when sessions were moved to the Stellenbosch University campus. To facilitate these meetings, each school nominated a staff member (e.g. teacher, secretary) to act as liaison between the school, NC, and adolescent counselee.

Fifty-three adolescents from four schools volunteered for the RCT during 2014, 12 of whom consented and met inclusion criteria (13–18 years old, PTSD diagnosis by independent evaluator). There were four active SLs during 2014.

### Nested study sampling

Data was collected in 2015 where all of the stakeholders who had been in the trial in 2014 [NCs (n = 6), SLs (n = 4), adolescent participants (n = 12)], were telephonically invited to participate in the qualitative study [22]. All were invited regardless of treatment arm or completion status in an attempt to draw on a broad range of experiences. Participants were informed that their feedback could provide valuable insights on improving the interventions and that their feedback, participation, or withdrawal would not affect their status in the RCT.

The first author, a clinical psychologist new to the study, conducted in person semi-structured interviews with the SLs (discussion schedule Appendix A), NCs (discussion schedule Appendix B), and adolescents (results provided elsewhere). The discussion schedule was used as a flexible tool to guide exploration of stakeholder experiences to inform the research team about the acceptability, feasibility, and impact [29–29] of the task-shifting interventions. Interviews ranged between 30 and 90 min. SLs and NCs were specifically questioned about their experiences of providing or coordinating treatment at school, perceived impediments to implementation, and recommendations for future intervention delivery. Interviews took place at Stellenbosch University Campus or at the representative school, as best suited to participants. One interview took place via video Skype.

### Participants

The six eligible NCs and four SLs were female. Three NCs declined participation on account of time constraints and one SL was unreachable. Table 1 provides a summary of background information for the NCs who consented (identified by pseudonyms starting with N) and the SLs (identified by pseudonyms starting with T since most were teachers). Unfortunately the age of participants was not collected during the interview.

**Table 1:** Demographics of Participants

Pseudonym	Background
‘Natasha’ (N1)	White Afrikaans nurse from out of town with 6 years psychiatry experience
‘Natalia’ (N2)	White Afrikaans nurse with 30 years nursing experience
‘Noleen’ (N3)	Black Xhosa nurse, working in psychiatric hospital since 2006
‘Theresa’ (T1)	Afrikaans coloured administrator, basic counselling training at local NGO (*Lifeline*)
‘Tina’ (T2)	Coloured Afrikaans teacher, youth work experience, desires to study psychology
‘Thandi’ (T3)	Black Xhosa teacher, no known counselling background

### Analysis

The first author and a research assistant doubly transcribed the audio recordings to enhance accuracy, particularly in view of the dual language of the recordings (English and Afrikaans). Afrikaans transcripts were translated into English. Due to resource constraints, only nurse transcripts were member checked for the accuracy of transcription. The analysis process followed five steps:

The first two authors independently read through all the transcripts of all the stakeholders and identified 36 coding units.

Following discussion between authors 1 and 2, the 36 coding units were collapsed into six overarching themes.

The first author used Atlas.ti software to code the data into coding units and identified themes.

The second author re-read the transcripts and included any contradictory and/or outstanding data.

Upon further analysis and discussion, themes were re-named and re-grouped based on overlap that emerged among the three stakeholder groups (adolescents, NCs, SLs).

i. Adolescent experiences of accessing treatment (described elsewhere)
ii. Adolescent and NC perspectives of treatment efficacy (described elsewhere)
iii. NC and SL identified catalysts and impediments to task-shifting strategy

In this manuscript, we elaborate on NCs and SLs descriptions of the catalysts and impediments (and accompanying recommendations) they encountered during these task-shifting interventions. This narrative is made up of three sub-units: (i) personal care (coping), (ii) community care (logistics of the community setting), and (iii) collaborative care (importance of networking).

### Ethics

The study was approved by Stellenbosch University Human Research Ethics Committee (N12/06/031). Participants provided written informed consent for participation in recorded interviews. Transcripts were de-identified and stored in a locked research office with the audio recordings. Participants received a ZAR 50 grocery voucher for their time and transport costs.

## Results

The results are presented in three clusters: Personal Care, Community Care, and Collaborative Care.

### Personal Care

#### Impediments

Mastery of the interventions was regarded as *“rather tough”* (‘Natasha’). The PE-A treatment manual was complex and nurses often requested verbal explanations as a memory refresher. ‘Natalia’ felt like she was *“hyperventilating”* the first time she recorded a session for supervision.

Nurses approached the adolescents with grave responsibility. Participants were minors who *“puts his trust in you as the adult”* (‘Natalia’). Nurses were required to function independently from the customary setting where they were typically acting as a junior member in a multi-disciplinary team and having *“shared responsibility”* (‘Noleen’)—where *“you are a supportive person”* (‘Natasha’). Nurses wrestled with the notion of *“keep[ing] your professional distance”* (‘Natalia’) during counselling. This challenge was amplified by not being prepared *“for what the children would actually tell you”* (‘Natasha’), knowing that the child would be returning to a *“dysfunctional system”* (‘Noleen’), being *“acutely aware of their circumstances”* (‘Natalia’), and feeling guilt about *“leaving them again”* (‘Noleen’).

*After I’ve realized what they’ve gone through… you want to see them succeed… Like, a mother. You give birth to a child because at the end of the day you want them to be independent … I think in a study or it was just a neighbour’s child or whatever I would have done something. But I was also aware of the professional boundary. Because I was a professional in that space… That sense of wishing to do more and thinking that the child is going to that situation. I was overwhelmed. I was really overwhelmed by that… But you also know that you are not a mom… But it’s like, who is going to do this? (‘Noleen’)*

The struggle was not exclusive to the NCs, but also to the SLs. ‘Theresa’, now retired, described how challenging it was for her to know that there are children at her school who are going through hard times:

*When I started there I, yoh, took everything home with me. Everything. Until the school psychologist told me: you cut off! Because one night I was in such a state about a child that I started to shake. And my husband said: you can’t carry on like this. You must cut off! Then I said: but how do you sleep if you know that the child might not be safe? So I have had children at my home too, who slept there to be a safe haven. (‘Theresa’)*

#### Catalysts

Aside from 30 years of nursing experience which *“teaches you to keep your distance…. [otherwise] every time that I was there I would have cried with that child”* (‘Natalia’), supervision helped ‘Natasha’ to learn to *“not make [what the adolescent tells me] my own… Supervision is really important else you sit with those feelings.”* Supervision was also an opportunity to receive peer support and feedback in addition to guidance from an experienced trainer. It extended beyond the practical components of treatment adherence (review of video recordings of sessions) and incorporated support to aid NCs coping with traumatic stories.

The supervisor was easily accessible and attentive to ‘Natasha’s distress and individually taught her *“how to regulate my breathing and how to manage my own anxiety… that I don’t freak out while I am busy with [counselling].*” Additionally, supervision also provided ‘Noleen’ with the reassurance that adolescents would be appropriately referred if needed and that fieldworkers “*won’t just cross [their] arms and do nothing.”*

However, ‘Natalia’ noted that whilst she appreciated the supervision, she found it challenging to receive the supervision in a group setting. *“I [had] things that I can say but I keep it to myself… I know it is my own fault… Because … everyone was actually so extroverted”* (‘Natalia’).

#### Recommendations

NCs initially resisted supervision as they had no comparable previous experience. ‘Natasha’ has a hometown supervisor with *“no comprehension of psychiatry”* or the impact it can have on a person when *“you take all these things with you”* (‘Natasha’). *“They will just tell you to phone ICAS [Independent Counselling and Advisory Services]. I don’t want to… I want to see the person that I am talking to”* (‘Natasha’). Receiving face-to-face supervision allowed ‘Noleen’ to overcome her previous sensitivity to criticism, something she wished she was assisted with during her training as nurse:

*At the end sometimes I wouldn’t like that if my recording is stopped before time because I want … [the supervisor] to hear the whole thing, not just a bit … For the first time in 6* *years working in a therapeutic ward, I am not ashamed … to have people observing me behind the mirror because, I feel because I received the supervision. I am able to communicate … in a right way.*

Having witnessed the benefits of supervision in their personal development, they emphasized that nurses would be less resistant to supervision if it was incorporated throughout their nursing training – making it a norm rather than an exception.

## Community Care

### Transport

#### Impediments

NCs experienced the process of traveling to the schools in unfamiliar areas as *“the most negative thing”* (‘Natalia’) and *“a nightmare”* (‘Natasha’). For ‘Natalia’ *‘a big negative thing [was the] distance… because this is not an area where I live.”* ‘Natasha’ described her concern of driving to a school in a crime-riddled area: “*I stand out like a sore finger… This little car with the white woman into [this neighbourhood] …. I was rather anxious about that… I didn’t know if I would come out alive on the other side.*”

#### Catalysts

Although the driving was very stressful, it was also highly appreciated. ‘Natalia’ found it helpful to see the adolescents’ circumstances to understand her counselee better. Furthermore, ‘Tina’ (NC) was really pleased about the service delivery, including providing transport if the sessions were conducted at the university campus.

#### Recommendations

NCs recommended that counselling continue to be provided in school settings, highlighting that they would *“feel safer”* (‘Natasha’) if a fieldworker showed them a safe route the first time. ‘Natalia’ recommended that nurses be sent to the schools in areas that they were familiar with or that schools be allocated to counsellors according to the distance from their homes.

### Communication

#### Impediments

Recruitment took place during school assemblies or Life Orientation lessons which are compulsory school lessons dedicated to self- and career- development. Although those classes were smaller, it *“was still not intimate enough … Children are smart. They can read body language*” (‘Tina’). The teachers’ criticism of the task-shifting interventions was that they did not receive feedback on the participants’ progress. They highlighted the urgency of providing continued interventions or suitable alternatives (e.g. referrals and follow-ups).

#### Catalysts

Teachers concurred that the Life Orientation classes were more intimate and thus more effective for recruitment. Other efforts to maximize privacy included refraining from talking to the adolescents *“about their problem; what they experienced… because I am sitting with a full classroom when the child comes in here… I don’t want others to hear”* (‘Tina’).

#### Recommendations

‘Thandi’ recommended including parents in the recruitment process to *“explain how the counselling would help their children”* as adolescents *“are even afraid to tell them.*” In accordance with the need for holistic interventions, teachers urged waiver of age related criteria: *“We would really appreciate it if the project would cover them as well as long as they are part of this High School community”* (‘Thandi’). In fact, there is a *“very large need [to include primary schools]. Because that child comes with that problem from primary school*” (‘Tina’).

Having one person coordinating all the referrals of potential participants was found to be helpful since at least one person at the school had *“an understanding of what it is that we are coming to do … [and made an] effort for you to get the venue and get the children together”* (‘Noleen’). Feedback could be provided to this coordinator through a short report enabling the school to follow up and refer adolescents post-trial and assist the school to assess *“the success of whatever happened”* (‘Thandi’).

### Coordination of sessions

#### Impediments

At some schools, NCs could *“liaise with the teacher”* whilst at other schools *“there is not even anyone if the child is emotional… [to] ask that at least they can just look in for her”* (‘Noleen’). ‘Thandi’ sometimes forgot *“it’s Friday… and then maybe find out at the last hour that the hall is being used. Then, yoh!”* NCs admitted that it was frustrating when venues were unprepared because it made them look *“unprofessional”* (‘Natalia’) or like they didn’t follow *“certain processes”* (‘Noleen’). Nevertheless, SLs experienced the NCs as *“very understanding. They would be patient and allow me to do whatever is needed to be done at that moment”* (‘Thandi’).

Although ‘Thandi’ made the library hall (and corners of the hall for multiple sessions at the same time) available, *“we do not have enough rooms.”* NCs, SLs, and the first author’s field notes emphasise that the school venues were not the most suited for therapy. Sessions were characterised by interruptions such as *“a knock at the door… the telephone that rings every time”* (‘Theresa’), lack of private space, and noise (construction and traffic noise). Whilst this was alleviated by fieldworkers collecting participants for their therapy appointments to take place at university, it added to the burden of time- and resource constraints.

#### Catalysts

‘Natalia’ described herself as one of the *“lucky ones”* because she got the same venue every time which enabled her to acclimatise. Nevertheless, she preferred counselling at the *“quiet”’* university when the school setting was unavailable (e.g. school holidays). SLs made use of creative strategies to ensure that participants attended counselling sessions. During term time, *“we didn’t tell them what time the [counsellors] are going to come*” (‘Theresa’) because else they would skip school that day. During exam times ‘Theresa’ would remind both the student and supervising teacher, to ensure that the participant was at the appropriate venue once the bell had rung. ‘Tina’ tried to prepare a day ahead to keep everything organized. These preparations were made amidst teaching responsibilities and included securing approval from the principal, coordinating a venue, receiving the fieldworker or counsellor, and accompanying them to the venue.

#### Recommendations

Coordination efforts worked best for teachers and fieldworkers using WhatsApp (‘Theresa’).

## Collaborative care

### Impediments

Throughout the interviews it became clear that community members did not have access to psychologists, or suitably trained nurses, teachers, or community resources; which had been highlighted in previous research [32–32]. In ‘Noleen’s’ experience people are *“chase[d] away”* at private and public institutions because nurses do not get *“intensive psychiatric skills”* during their four-year training resulting in*” a lot of damage … being done … to people by nurses who don’t have the skills*”. Teachers are also limited in their scope—*“you can’t do the work of psychologist. You can cause major damage”* (‘Tina’). ‘Thandi’s school once facilitated a forum where students could talk about their problems. Despite honest and emotional sharing, *“it ended there. We couldn’t help those kids.”* ‘Thandi’ reported that pressures to complete the curriculum, assessments, administrative tasks, and manage the sheer number of students in a class *“gets so frustrating… You don’t even have a chance to ask if there is a problem or something.”* External stressors also affected nurses’ capacity to engage in the project. Part of *“having my own load”* (‘Noleen’) included academic pressures, studying away from home, and not having their own transport. This pressure is exacerbated due to lack of staff, for example in ‘Natasha’s home town they *“don’t have a Multidisciplinary Team (MDT). It’s just me … You are the psychiatrist… You are the psychologist… You are the social worker. You are the whole team. So you do everything.”*

As the department of education does not provide funding for psychologists to visit the school weekly, ‘Theresa’ asserts the importance of collaboration with community resources. Teachers expressed frustration at the lack of teamwork. Positive initiatives fail, for example, *Power Child* is no longer offered at ‘Thandi’s school because staff are unwilling *“to take the responsibility… Once you come up with an idea it is your baby”* (‘Tina’). Similarly, although ‘Noleen’ was convinced about the value of prolonged exposure therapy, she *“won’t be able to do it at work … I don’t have the power to convince my team that [it] is something that can help the patient.”*

NCs and SLs expressed frustration that *“sometimes people work against you”* (‘Noleen’), *“breaking down where I am trying to build … your hands are tied behind your back… you feel like you are against the wall”* (‘Tina’). ‘Noleen’ described one particularly distressing experience where she was trying to provide counselling to a participant:

*I didn’t have space and I was given someone else’s space. And then he came. He demanded to use his space … This child who had been absent to school. He wants his office to work… It’s like, he is not even interested why this child has not been coming to school, or if maybe I am trying to do something that is going to help the child … I would feel like, you know, I am failing this child… I was thinking a lot of things the child might think: why should I carry on with this thing because even the teachers they don’t even see any value in what is going on.*

### Catalysts

In contrast, there are times where *“[staff] realizes that there is a need… and they are willing to help as far as they can even if it is only to supervise your class; then they have done something”* (‘Tina’). Throughout the interviews teachers provided rich evidence of resourceful networking (Table 2) to identify opportunities for their students.

**Table 2:** Community resources accessed by the schools

Resource	Description and success
School staff	‘Tina’ and ‘Theresa’ received counselling training at a youth centre and *Lifeline* respectively, but both were too busy to implement their skills.*”It’s not only a parental responsibility, the child spends a lot of time at school so the school needs to take up some responsibility and take care of the children”* (‘Thandi’)
Student development centre	‘Thandi’ explained that the school makes venues available during lunch time and after school for community programs. Although initially stigmatized, now students *“bring a friend”* (‘Tina’). At these centres, students can bring their social, learning, and emotional problems, or even use it as a study venue (‘Theresa’)
Lay counsellor	A community lay person does workshops at ‘Theresa’s’ school. A clergy member initially worked for free to *“liaise with social workers and psychologists to collect the child’s whole background so that they can deal with it”* (‘Tina’). This volunteer now *“has a small office, tiny, and she earns a salary… student governing body”* (‘Tina’)
Community organizations	*African Tycoon* and *Power Child “offer computer literacy, computer training, sport … a plate of a full meal”* (‘Thandi’) while *Read and Write Solutions* takes parents from the community to visit schools to help the children to read and do math. *Molo Songololo* had powerful impact in the community where one graduated student is now in Amsterdam working for *LoveLife* and another is a chef while *We Can* teaches students *“how to start your own business”* (‘Theresa’)
University	‘Theresa’ reported collaboration with local universities sending remedial student teachers and staff serving at the *Bathuthuzele Care Centre*
Social worker	‘Theresa’ telephonically consults a social worker where to refer students (e.g. NGOs).*”There is one social worker … [She] is … busy… Has got her own work schedule… Parents just don’t have time to consult”* (‘Thandi’)

### Recommendations

Nurses and teachers concurred that although teachers could acquire necessary skills, it may be easier to train nurses. ‘Theresa’ said: *“You need somebody from the community to run it. You can’t have a teacher run it.”* Teachers have *“their own responsibilities”* (‘Noleen’) and their psychology studies are not *“that in depth”* (‘Thandi’). In contrast, nurses have *“an understanding already of psychiatry”* (‘Noleen’) and acquiring counselling training can make them central to the successful identification and *“hold[ing]”* of persons with emotional problems (‘Noleen’) resulting in *“less need for medication”* (‘Natasha’).

Furthermore, ‘Noleen’ recommended that teachers understand the project and see it as part of their job (not something additional), perhaps through collaboration between government departments of health and education. She added that even if all the nurses at the various community clinics do not have the necessary skills, it would be helpful for at least one staff member to have some counselling skills to *“identify what are the emotional problems, mental health problems, and all of that, and then intervene when it’s necessary”* (‘Noleen’).

## Discussion

Based on these qualitative descriptions of the experiences of nurse counsellors delivering psychotherapeutic interventions under supervision and the perspectives of liaisons at the schools where most of the interventions were offered, it appears that providing treatment within the community was well received in spite of the many impediments mentioned. In fact, SLs expressed a strong urging that children not be excluded from the interventions going forward due to age-related criteria.

### Personal care

The underlying maternal nature of the SLs and NCs struggles are evident. In a country where the number of child-headed households is high (54,000 households) and the majority of children do not live with both parents (66%) or either of their parents (20%) [33], children often lack the *“adult”* (‘Natalia’) and *“supportive person”* (‘Natasha’) they need. It is not surprising that the adolescents so freely shared their experiences and put their trust in the adults who showed an interest, as was also indicated in previous research [34]. This highlights the importance of providing teachers and nurses with the necessary support and training in order to assist them with keeping professional boundaries. Although initially resistant to supervision, it was central to the success of the intervention and provided an excellent vehicle to support the NCs while functioning away from the MDT. It may be advisable to include a one on one individual supervision session to navigate personality differences and counteract missing valuable feedback. Whilst valuable, supervision during training may not be feasible due to resource constraints at training institutions [30, 35]. NCs reports of anxiety initiating the delivery of the interventions highlights the importance of ensuring that intervention manuals are as simple as possible. When employing task-shifting, enough time should be allocated to training not only to ensure successful delivery of the intervention, but build confidence in the service providers [8, 12].

### Community care

Logistical limitations included transport, communication, and securing suitable times and venues for sessions. NCs took pride in their work and felt frustrated when the logistical arrangements were not done as professionally as they would have liked. In providing community based care, service providers will have to become more flexible to allow for sustainable service delivery. One way to help them accept these practical challenges (e.g. getting lost, miscommunication, appointments being late or not kept, trouble securing venues), could be to reduce their fear of being reprimanded for not following the red-tape associated with the service. Supervision and support could help service providers to navigate their anxiety of being perceived as unprofessional or ill-prepared. With Cape Town being labelled as the most congested city in South Africa in 2016 (http://www.tomtom.com), it is not surprising that the nurses found driving to and from the schools daunting. Whilst not necessarily mentioned by the participants, allocating counsellors according to the distance from their home will also make the intervention more cost- and time-effective.

### Collaborative care

SLs were master networkers able to identify a myriad of resources available to their students despite acute resource shortages in mental health care in South Africa. Both SLs and NCs were optimistic about the task-shifting interventions and recommended training more nurses. Unfortunately, imperfect collaboration will remain a potential barrier to successful scale-up unless there is greater teamwork between government departments of health and education.

## Conclusion

This study adds to the small body of literature describing the experiences of the non-specialist health workers providing task-shifting mental health care. This study is unique in the way it not only addresses the experience of the NCs, but also the SLs who acted as a link between the intervention and the adolescents in a community based setting.

Limitations include the lack of individual follow up interviews restricting opportunities to clarify content and guarantee data saturation. In addition, stakeholders who declined participation may have provided stronger criticism of the RCT.

Policy makers and clinicians should heed the following practical recommendations for scale-up:

I. Provide counselling training to nurses as part of the undergraduate training program and incorporate supervision in the process. Identify suitable nurses and distribute them throughout communities to act as liaisons.
II. Assign at least one staff member at each school and guarantee time off for effective coordination, referral and feedback. A quiet, private venue should be made available.
III. Continuation of treatment at schools beyond the RCT. Nurses will need support with transport. Teachers and coordinators can communicate most effectively using a mobile phone application such as WhatsApp.

Our findings provide support for the capability of task-shifting psychotherapeutic interventions for PTSD in a school setting while affirming the importance of supervision [12]. Future studies should include follow up interviews to ensure data saturation. We also recommend optimising the use of existing resources and the potential of nurses to receive training in these two interventions.

## Appendices

### Appendix A: Interview Schedule for School Liaisons (SLs)

Activities

1. How did you become involved in this project?
  - What do you know about this project?
2. What was your role in this project?
  - Did you choose it?
  - Were you assigned to this role?

Acceptability (Like/dislike)

3. What did you like/dislike about this role?
  - Popular?
  - Help the children?
  - Extra work and pressure?
  - Resented being forced?
4. What did you think about the project when you first heard about it?
  - A good idea? Bad idea?
5. How did your opinion change/stay the same during the course of this project?

Feasibility (barriers/facilitators)

6. What do you think made the recruitment at school un/successful?
  - How many scholars from your school?
  - Should there have been more? Why?
  - How do you identify those children?
  - What would have made it possible for them to get this kind of help?
7. What are the practical implications and challenges of arranging for scholars to receive treatment at school?
  - Venues?
  - Time?
  - Contact with the team?

Impact (pros and cons)

8. What effects could you see as a result of the intervention?
  - Did it help or not?
  - Stigma?
  - Interaction with other scholars?

Suggestions

9. Why would you dis/encourage mental health interventions at your school in the future?
  - And for other schools?
  - For your own child?
10. What suggestions would you make to ensure that this project’s success and sustainability?
  - If you were in charge of the department of education, would you recommend that this project gets rolled out on a larger scale?
  - What changes would you make?

### Appendix B: Interview Schedule for Nurse Counsellors (NCs)

Activities

1. What was your experience of the many different activities you were involved in?
  - Like, dislike
  - Barriers, facilitators
  - Pros, cons
  - Training and supervision
  - PE-A and SC
2. What was it like to counsel traumatized teenagers?
  - What helped you to cope?
  - What was difficult?
  - What surprised you?

Acceptability (Like/dislike)

3. What intervention (parts of the intervention) did you prefer? Why?
  - Supportive counselling?
  - Prolonged exposure?
  - Recruitment?
  - Going to schools?
4. What was it like to receive group supervision? Especially if you did not get to provide both treatments?
  - How was it different?
  - What was hard?
  - What were you relieved about?

Feasibility (barriers/facilitators)

5. What are the rewards and challenges of being part of this study?
  - What was your highlight?
  - Lowlight?
6. How did a contact person at school influence the success of this process?
  - How can one identify such a person?
  - What did the person do?

Impact (pros and cons)

7. What would make you feel more equipped to do these interventions?
  - Manual?
  - More freedom?
  - Practice?
8. Why would you (not) continue with task shifting as a future career?
  - What kind of feedback do you get from others when you tell them you are part of this project?
9. What are you still implementing from your learning during this project?
  - How did you grow? How are you different now?

Suggestions

10. What suggestions would you make to ensure that this project’s success and sustainability?
  - If you were in charge, how would you make it better?

## Abbreviations

RCT: randomized controlled trail
PTSD: posttraumatic stress disorder
PE-A: prolonged exposure therapy for adolescents
SC: supportive counselling
NC: nurse counsellor
SL: school liaison
MDT: multi-disciplinary team
